# Genetic Variability and Admixture Zones in the Italian Populations of Turkey Oak (*Quercus cerris* L.)

**DOI:** 10.3390/life13010018

**Published:** 2022-12-21

**Authors:** Bruno Bertolasi, Luisa Zago, Lorenzo Gui, Piero Cossu, Isabella Vanetti, Silvio Rizzi, Marta Cavallini, Gianluca Lombardo, Giorgio Binelli

**Affiliations:** 1 Centro Nazionale Carabinieri Biodiversità (CNCB), 37020 Peri, Italy; 2 Dipartimento di Medicina Veterinaria, Università degli Studi di Sassari, 07100 Sassari, Italy; 3 Dipartimento di Scienze Teoriche e Applicate (DISTA), Università degli Studi dell’Insubria, 21100 Varese, Italy; 4 Dipartimento di Biotecnologie e Scienze della Vita (DBSV), Università degli Studi dell’Insubria, 21100 Varese, Italy

**Keywords:** *Quercus cerris* L., microsatellites, genetic variability, conservation, recolonisation

## Abstract

The Turkey oak (*Quercus cerris* L.) is widely distributed in Italy, where it is the ecologically dominant oak on sandy and acidic soil. In this work, we analysed 23 natural populations by means of eight SSR (microsatellite) markers, to obtain the first synthetic map of genetic variability for this species and to study its dispersion during the Holocene, due to the possibility that at least one refugium during the Last Glacial Maximum was in Italy. The analyses showed a good amount of genetic variability together with fair differentiation between populations, as indicated by *F_ST_* = 0.059. A Bayesian analysis of the amount of admixture among populations revealed the presence of four putative gene pools of origin and a rough subdivision of the populations according to their geographic location, as confirmed by the spatial analysis. No evidence for the existence of putative refugial populations was found; however, this study paves the way for the planning of conservation strategies also with regard to the relationship between Turkey oak and other oak species in Italy.

## 1. Introduction

The oak is arguably the most famous amongst trees. Across Europe, oak trees have been revered as sacred to the major divinities of many cultures; the Latin name itself, “*robur*”, means “strength, robust and firm”. However, aside from their importance for human culture, the different species of the genus *Quercus* are also of great interest for the study of the evolution of trees. In fact, it is commonly recognised a “complex of species” belonging to *Quercus*, due to the occurrence of a fair amount of hybridisation between the different species. This has been demonstrated for pedunculate and sessile oak, *Quercus robur* and *Q. petraea,* respectively; [1,2,3] for *Q. ilex* and *Q. suber* [4,5,6,7,8], *Q. petraea* and *Q. pubescens* [9,10]; and for New World species such as *Q. crassifolia* and *Q. crassipes* [11]. In the last decade, both hybridisation and hybrid fertility have been demonstrated by means of genetic markers between *Q. coccifera* and *Q. ilex* in Spain [12]. Interspecific gene flow is particularly evident when looking at the plastid genome of the chloroplast: different evidence supports the hypothesis of hybridisation again between pedunculate and sessile oak (see [13] for a review). The most spectacular case is that of a forest in west–central Romania, where four species of oak (*robur*, *petraea*, *pubescens* and *frainetto*) all interbreed [14]. In the latter study, a fifth *Quercus* species is present but apparently not intermixing—this is *Q. cerris*, the object of our study.

Among the different oaks, scarce attention from a genetic point of view has been devoted to the Turkey oak, *Quercus cerris*, a species of still uncertain provenance despite its common name and whose habitat is essentially limited to the Italian and Balkan peninsulas (Figure S1), with a few populations scattered in Southwestern France and Anatolia [15,16]. This is a tree of great dimensions and majestic standing, easily mistaken with *Q. petraea* from a distance [17] and often growing in mixed stands with *Q. robur*—a fact that has led to debate over the existence of *Q. cerris* × *Q. robur* hybrids. Very little is known about its diffusion during the Last Glacial Maximum and its re-colonisation routes during the Holocene unlike the extensively studied *Q. petraea*, *Q. robur*, *Q. pubescens* and other representatives of the so-called “white oaks”. The only palynological data for *Q. cerris* indicate a very circumscribed area in the southwest of Turkey as refugium [18].

In Italy, this species is vastly distributed throughout the whole peninsula and Sicily, but not in Sardinia, and its ecological dominance is widely recognised also in toponomastics: many cities and villages bear the name “Cerro” or “Cerreto/a” in Italian, after the species and standings, respectively.

The aim of the work presented in this paper is to define the first synthetic map of the genetic variability of Turkey oak in Italy for two purposes. Firstly, for the conservation of the species, whose availability is more and more threatened by human activities coupled with its fast growth habit on sandy and acidic soil [19,20] and its great water request. These factors make the species sensitive to climatic change, as stressed by the very hot summer of 2007, which caused the loss of one third of the foliage of *Q. cerris* and *Q. pubescens* in central Southern Italy. Secondly, we are interested in the retracing of its diffusion during the Holocene because of the possibility that at least one refugium during the Last Glacial Maximum was in Italy. The interest in this second aspect resides in the fact that today’s diffusion of Turkey oak is roughly only half of the available area for the species under a climatic point of view [21]. 

## 2. Materials and Methods

### 2.1. Plant Material and DNA Extraction 

**Figure 1:**
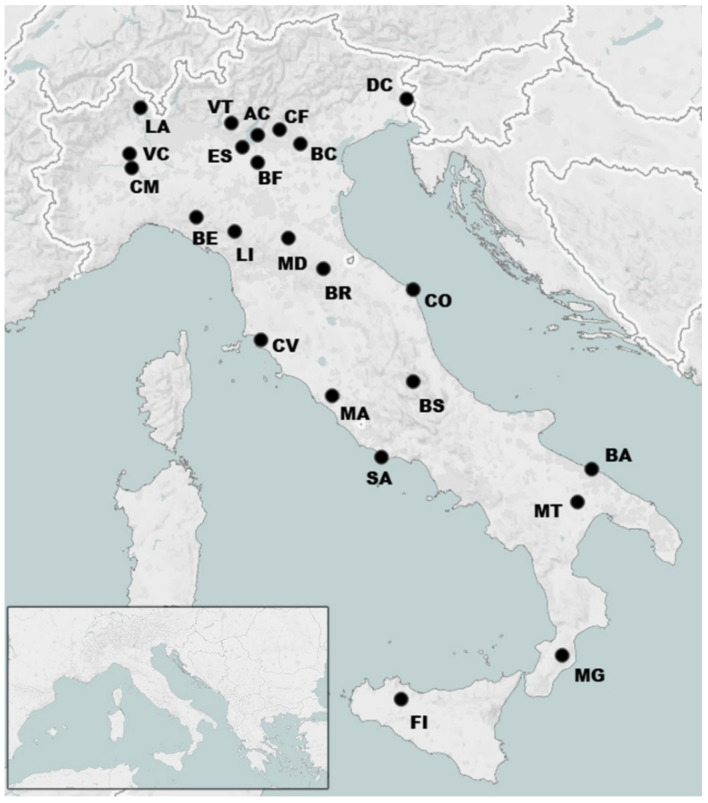
Schematic map of Italy showing the geographic localisation of the populations of *Quercus cerris* studied (see also “Map position” in Table 1). Inset shows the Mediterranean basin.

To assess the genetic diversity of the Turkey oak (*Quercus cerris* L.) in Italy, we sampled across most of the species’ Italian distribution range, discovering, in the process, at least one natural population in an area where the presence of *Q. cerris* was not previously described. In particular, we sampled 22 populations along the Italian peninsula and one in Sicily (Sardinia was excluded from the sampling plan since this species is not naturally present) throughout the species’ Italian distribution. Interestingly, a number of our sampled areas had unexpected *Q. cerris* distribution and represent a new finding in the Italian natural history of the species. These lie outside what is considered its ‘native distribution’ [15,16] and were collected in Bari (Apulia), Vercelli (Piedmont) and Ficuzza (Sicily) (Figure 1 and Figure S1). A total of 809 trees were sampled, with an average of 35 trees per population. Populations were selected based on historical data about forest management in order to exclude populations of uncertain origin or the ones that underwent artificial reforestation. Only healthy adult trees with clear morphological traits were included in this study, also taking into account the presence of mixed stands with other oak species and the possible occurrence of hybrids. Morphological traits used for species identification were mostly based on leaf shape (leaves are lobed with a variable number of triangular lobes), leaf surface aspects (glossy upper surface), bark aspects (vertically deeply furrowed and dark grey) and acorn aspects (hairy acorn cup) [17]. The geographical position and coordinates of the populations studied are reported in Figure 1 and Table 1, respectively. 

For every single tree, 4–5 young leaves, collected mostly in spring to early summer, were stored at room temperature in 10 mL of 95% ethanol until DNA extraction. Approximately 1 cm^2^ of re-hydrated leaf tissue was used for DNA extraction while the other leaves were frozen at −20 °C. DNA was extracted with the use of DNeasy™ Plant Mini Kit (Qiagen S.r.l., Milan, Italy) according to the manufacturer’s instructions.

### 2.2. SSR Markers

After screening several dinucleotide and trinucleotide simple sequence repeat (SSR) markers available from the literature, eight heterologous markers were selected: MSQ4 and MSQ13 developed in *Q. macrocarpa* [22]; QpZAG9 and QpZAG110 developed in *Q. petraea* [23]; QrZAG4, QrZAG7, QrZAG20 and QrZAG96 developed in *Q. robur* [24]. These markers yielded a simple amplification pattern in *Q. cerris* and mapped onto different chromosomes in a comparative genetic map for *Q. robur* and *C. sativa* [25]. The primer sequences and annealing temperatures (T_a_) for each SSR are listed in Table 2. PCR amplifications were carried out in a total volume of 20 µL with the following mixture: 11.6 µL distilled water, 4 µL 5× PCR buffer, 1.6 µL MgCl_2_ 25 mM (final conc. 2 mM), 0.4 µL 10 mM dNTPs (final conc. 0.2 mM of each dATP, dGTP, dTTP and dCTP), 0.4 µL 10 µM of each primer (final conc. 0.2 µM), 0.1 µL 5 U/µL GoTAQ^®^ Flexi DNA Polymerase (Promega Italia, S.r.l., Milan, Italy, final conc. 0.5 U/tube) and 1.5 µL genomic DNA 7.5 ng/µL. 

Amplification profiles of various loci included an initial denaturation step (94 °C for 2 min); followed by 35 cycles of 94 °C for 20 s, T_a_ for 20 s and 72 °C for 30 s; followed by a final step at 4 °C.

Genotyping was carried out on a MegaBACE™ 1000 automatic sequencer (Amersham Biosciences, Amersham, UK) in multiplexed runs (three markers each) and with the use of ET-Rox 400^™^ as the molecular standard (General-Healthcare, Chicago, IL, USA). Different amplicons were marked using fluorescent-labelled primers (5′ end marked with one of the following fluorochromes: 6-FAM, HEX and TAMRA from Sigma-Aldrich S.r.l., Milan, Italy). Amplicons were scored using MegaBACE™ Fragment Profiler software version 1.2 (Amersham Biosciences).

### 2.3. Genetic Analysis 

First, we estimated at each locus, for all populations, allele frequencies and observed (*H_o_*) and expected (*H_e_*) heterozygosity. Deviations from the Hardy–Weinberg equilibrium were assessed by Fisher’s exact test using a Markov Chain algorithm [26]. Genotypic disequilibrium between pairs of loci was also tested within populations by Fisher’s exact test. Genetic differentiation within and between populations was studied by Weir and Cockerham’s [27] estimators of *F*-statistics. In particular, *F_IS_* estimates which proportion of the total genetic variation is due to a departure from the Hardy–Weinberg equilibrium at the population level and *F_ST_* estimates the amount of total genetic variation due to differentiation between populations. The *F_ST_* analogue for microsatellites *R_ST_* [28] was also used; we included the size of differences between the alleles in the differentiation estimates. The matrices of pairwise *F_ST_*/(1 − *F_ST_*) and log-transformed geographical distances between populations underwent a Mantel test [29] to assess isolation-by-distance, interpreted as the presence of migration–drift equilibrium between populations. The software packages used to analyse the genetic data were genepop 4.0 [30], genetix [31] and rstcalc [32]. 

Lastly, we used the spatial Bayesian clustering algorithm implemented by TESS 2.3 [33] to reveal the presence of differentiated gene pools in the dataset. In addition to individual multilocus genotypes, the method uses information on geographic location to estimate admixture proportions. More specifically, hidden regression models with autocorrelated residuals are used to incorporate spatial trends (global effects) and spatial autoregressive processes (local effects) in the prior distributions on these coefficients [34]. Spatial Bayesian clustering has been shown to perform better than non-spatial Bayesian clustering [35,36] in the case of low genetic differentiation, as well as in the detection of clines and secondary contact zones. 

Because a single pair of geographic coordinates was available for each population, each individual was assigned a randomly generated geographic location centred on the coordinates of population of origin, within a narrow range based upon a standard deviation of ±0.01 degrees in latitude and longitude. Individual geographic locations were used to build up a spatial connectivity network based on Dirichlet tessellation and to compute a matrix of great-circle geographic distances among individuals. Great-circle distances were used as weights to shape the magnitude of the spatial neighbourhood effect.

To identify the most probable number of clusters and model options, we used an iterative approach. We first performed 20 runs, setting the number of clusters at *K* = 23 (the number of populations in this work) to assess the upper bound in the maximum number of clusters. As evidenced by Durand et al. [34], once the Q-matrix stabilises, no more clusters appear when increasing the number of *K*. According to these results, the software was run 10 times for each number of clusters ranging from *K* = 2 to 10. Both these steps were carried out under the no-admixture model with 25,000 iterations, of which 10,000 were discarded as burn-in and setting the spatial interaction parameter at 0.6. 

The choice of the most probable number of clusters fitting the data was based on the score of the Deviance Information Criterion (DIC), which is computed by TESS and represents the average model deviance penalised by the effective number of parameters used in the model [34]. Since better models are expected to have lower DIC values, we tested the hypothesis that the mean DIC values for a model with a given *K* were significantly lower than the model with (*K* − 1) using a series of one-tailed paired *t*-tests carried out with R 4.1.3 (One Push-up) [37]. The procedure stops when the null hypothesis of no-difference could not be rejected. Additionally, to choose among the retained models, we assessed the consistency of results among replicate runs for each *K* by computing the coefficient of similarity based upon the G’ matrix pairwise similarity statistics implemented by the software clumpp 1.1.2 [38]. A Greedy algorithm with 10,000 iterations was used to align runs and account for label switching. 

We carried out a further series of runs for the selected *K* under the admixture model with 50,000 iterations, of which 30,000 were discarded as burn-in. Since gene flow is expected to be spatially restricted due to the species’ limited dispersal capabilities, we aimed at comparing models accounting either for broad-scale (e.g., historical events as migrations) and fine-scale (e.g., isolation by distance) patterns or fine-scale patterns only. Hence, we ran both full regression models (linear degree of spatial trend and either BYM or CAR as autoregressive models) and models without a spatial trend (constant degree of spatial trend and either BYM or CAR autoregressive models), respectively. We carried out 10 replicates for each model and then ranked them according to the lower DIC values. An additional 100 runs were completed for the most likely model, of which the 10 highest likelihood runs were retained and their clustering results were averaged using clumpp 1.1.2 by applying the Greedy algorithm with 10,000 replicates [38].

The posterior spatial predictions were displayed on the geographic map of Italy using functions from the R 2.14.0 [37] packages Spatial, Raster and Fields. The extent of any genetic cluster was mapped by using a generalised least squares trend surface interpolation [39] of admixture proportions. Following Row et al. [40], we fit a zero polynomial (constant) trend surface regression with an exponential covariance function to the admixture proportions (psill = 1, nugget = 0) for each cluster. We used a range parameter of 3 decimal degrees (about 335 km) and extrapolated the trend over the study area at a resolution of 2335 km. 

### 2.4. Demographic Analysis 

Approximate Bayesian Computation (ABC) was successively used on the four ancestral gene pools computed by TESS to conduct a model selection analysis, which infers possible variations in demographic scenarios. These analyses were performed using diyabc v2.1.0 [41] following the two methodologies used by Barthe et al. [42]. The first method allows for the effective population size (*N_e_*) to vary freely; this makes it possible for any demographic event to occur. The second method instead compares two opposite scenarios—a single expansion event or a single contraction event; these are then analysed using logistic regression to confirm the demographic model computed using the first method. The PABC index (Barthe et al. [42]) was also calculated to provide an estimate of the robustness of the demographic models. Both aforementioned methods used were estimated using three different demographic parameters: T, number of generations since the simulated event (log-normal distribution, mean = 300; SD = 250); N_0_ and N_1_, the population size after and before the simulated event, respectively (*log*-normal distribution, mean = 1000; SD = 1000). The mutation model used was based on the GSM, Generalised Stepwise Mutation [43] using an average mutation rate μ (uniform hyperprior distribution, min = 10^−5^; max = 10^−3^) and a stepwise constant P (uniform hyperprior distribution, min = 0.1; max = 0.3). Chosen summary statistics chosen were the average number of alleles, average genetic diversity and variance of average allele size. A total of 10^6^ datasets were simulated for each scenario with the aforementioned variables. 

## 3. Results

### 3.1. Genetic Variability 

A total of 809 *Quercus cerris* samples were analysed using eight microsatellite markers, resulting in the identification of 265 distinct alleles. All studied loci are highly polymorphic: the number of detected alleles per locus across all the populations ranges from 20 (locus QrZAG96) to 40 (locus QpZAG9) with an average of 35 alleles/locus. No alleles were found to be fixed at any of the loci, nor did we find evidence that specific alleles were harboured by a given population. In general, the number of alleles/locus/population is remarkably constant, between 10 and 14 with the exception of populations VC (8 alleles), ES and VT (15 alleles). Genetic diversity, as measured by Nei’s heterozygosity (*H_e_*), is high (Table 1), with the only exception being locus MSQ13, displaying *H_e_* values as low as 0.03 in the BC and BE populations; all other loci have *H_e_* values in the range of 0.6–0.9. Average *H_e_* shows a more restricted range, with values between 0.65 (VC population) and 0.81 (MT population). 

The Hardy–Weinberg equilibrium was tested for all the loci and populations by testing the departure of *F_IS_* from zero under the null hypothesis. *F_IS_* values are significantly different from zero for at least four loci out of eight in all populations; two populations, MT and BS, have no loci in equilibrium. In most cases, deviation from the Hardy–Weinberg equilibrium was associated with positive *F_IS_* values, while negative *F_IS_* values were found in six cases only (two each for populations BR and VC; one each for populations BS and MD). 

### 3.2. Genotypic Disequilibrium 

The non-random association of the alleles at different loci, or linkage disequilibrium (LD), was investigated. Since the number of comparisons for pairs of loci with eight loci is 28 per population, we expect one or two false positives per population. When all populations showing only one or two pairs of loci in disequilibrium are removed, only nine populations display a significant departure from equilibrium at the 5% level: CM, BC, LI, MA, MG, BS, CO, BA and MD. Of these, the MA population is characterised by the presence of 10 pairs of loci in LD.

### 3.3. Genetic Differentiation among Populations

The genetic divergence among populations was measured using both *F_ST_* and *R_ST_* (Table S1). Their significance was tested by a permutation procedure: the vast majority of *F_ST_* and *R_ST_* values differed significantly from zero with only six exceptions (AC-CV, BF-LI, BC-MA, DC-SA, MT-LI and MT-SA) out of 253 for *F_ST_* and a few more for *R_ST_*. The maximum *F_ST_* value of 0.135 was found between the MG and LA populations, and the maximum RST value of 0.273 between the FI and CM populations. The MG population is noteworthy in the sense that it displays *F_ST_* values higher than 0.09 in 15 out of 22 comparisons and higher than 0.2 in 16 out of 22 for *R_ST_*. It is also to be noted that, overall, the pairwise *R_ST_* values are constantly higher than the respective *F_ST_* values. 

The overall genetic differentiation between populations was significant. By means of *F_ST_* = 0.054 (confidence interval at 95% results in 0.035 ≤ *F_ST_* ≤ 0.086), it was estimated that about 5% of the genetic variance can be attributed to differentiation between populations. The same procedures for *R_ST_* yielded an estimated overall *R_ST_* = 0.071, with a confidence interval at 95% of 0.070 ≤ *R_ST_* ≤ 0.104. The presence of correlation between genetic differentiation and geographic distance between populations was not evidenced by a Mantel test (P = 0.88, G = −1.136, Z = 0.81), indicating that the present distribution of genetic variation among the studied populations of *Quercus cerris* cannot be seen as the result of equilibrium between drift and gene flow.

### 3.4. Analysis of the Population Structure 

Because *Q*. *cerris* has not been studied yet in this regard in Italy, it is important to estimate *K*—the most probable number of ‘gene pools’ present in the data—in order to be able to suggest possible mechanisms that have shaped genetic variability and to recommend conservation strategies. This was achieved by applying the spatial Bayesian clustering method, as implemented by TESS, which allowed us to define spatially the region of most probable admixture of the alleles carried by each of the putative gene pools of origin. 

**Figure 2:**
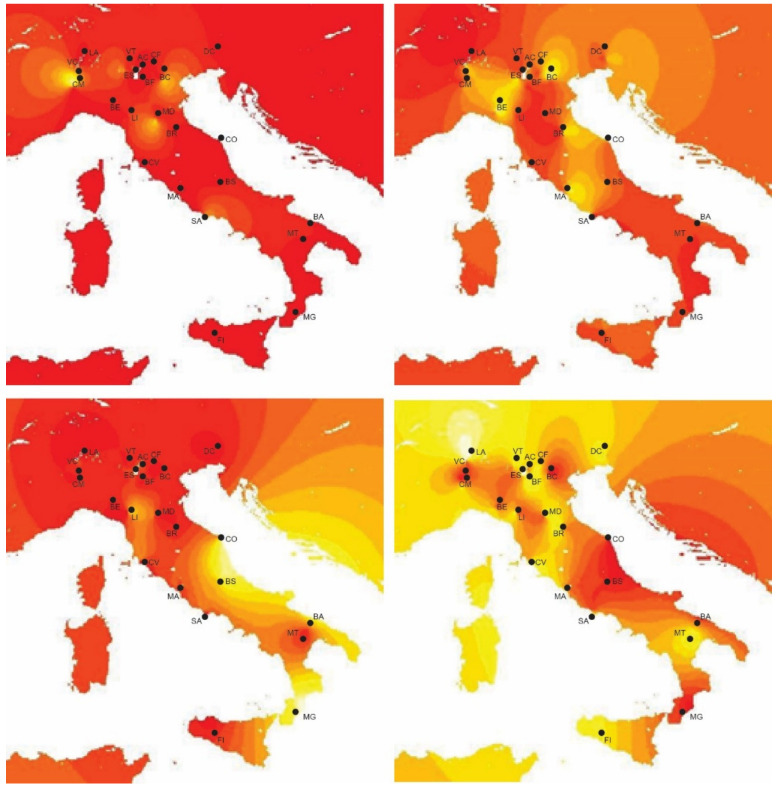
Admixture proportions of the four gene pools of origin projected on the Italian geographic map. The lighter the shade, the higher the admixture level. For an easier comparison with Figure 3, the top left panel shows the admixture proportions for the “red” gene pool, and the top right, bottom left and bottom right for the “blue”, “green” and “yellow” gene pools, respectively.

The estimate of *K* was based on the DIC criterion (see Section 2 and Table S2): the most probable result for the potential number of clusters that could have originated the studied populations is four. The proportions of admixture for each of the putative clusters of origin were spatially interpolated on the geographic map of Italy (Figure 2). 

**Figure 3:**
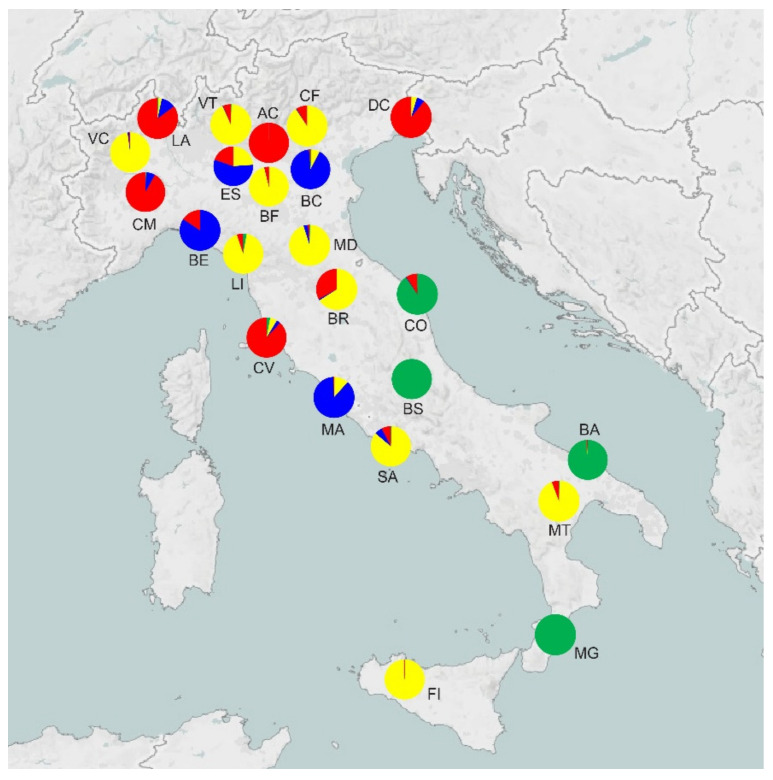
Schematic map of Italy. The proportions of ancestral gene pools of origin for each studied population are shown by pie charts. The colours match those of the bar chart of Figure S2.

Some aspects can be inferred by looking at the prevalence of each of the four ancestral genetic pools shown in Figure 3. Northern Italy appears to be the most admixed region, with the presence of three out of four genetic pools. The central and southern parts of Italy are more homogeneous, with the important presence of the fourth gene pool (green in Figure 3) apparently not admixed if not partially for the CO standing. Overall, the most important contribution is provided by the “yellow” gene pool, observed from north to south and with a peculiar presence in Sicily. The bar chart of Figure S2 in the Supplementary Materials displays the same information at the individual tree level for each population.

### 3.5. Population Demography

**Figure 4:**
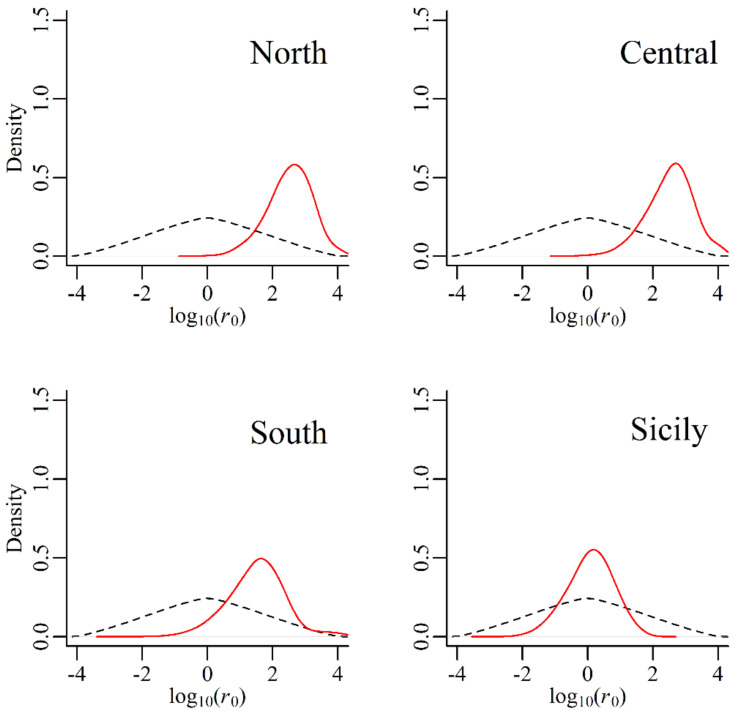
Density distributions of posterior estimates of the *r*_0_ ratio of present (*θ*_0_) and past (*θ*_1_) diversity population index (*r*_0_ *= θ*_0_*/θ*_1_) based upon the ABC “free variation” approach for the four homogenous clusters of *Quercus cerris*. Dashed lines: prior distribution; full lines: posterior distribution.

**Figure 5:**
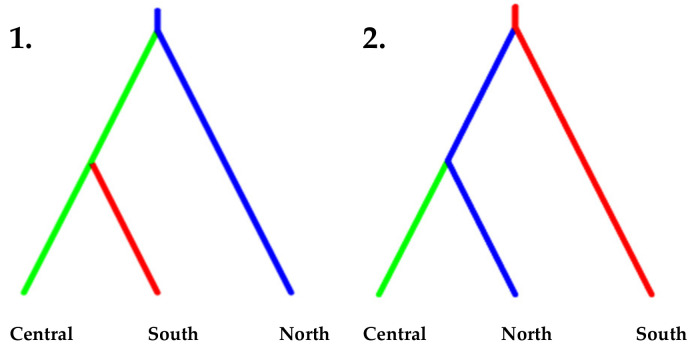
Coalescence model data. Expansion from north to south is the more probable event: Scenario 1 has a relative probability of 0.85 against the 0.15 of Scenario 2, as indicated by logistic regression.

The most probable demographic scenario (Table 3), given the “free variation” method, was estimated to be an expansion for all ancestral populations (coefficient *r*_0_ estimates of 424.44, 452.50 and 39.32) except the Sicilian/Pop4 (*r*_0_ = 1.53), who did not show a significant demographic change. The posterior coefficient *r*_0_ was also represented as distribution densities (Figure 4). These demographic events were estimated to have occurred between 628–4800 generations (Population 4 and Population 1, respectively). Using an estimate of 30 years for the Turkey oak generation time, we have estimates of 19–144 thousand years. The second method used logistic regression to estimate the two opposite scenarios (Figure 5). These results further confirmed the previous method with expansions occurring in the first three ancestral populations and a non-significant estimate of 0.53 (95% CI 0.50–0.55) for the fourth population. For each proposed event, the PABC index was computed based on the combined probability of type I and type II errors (Supplementary Material Table S3). Finally, the three ancestral pools with the most significant demographic change were grouped, and the two colonisation scenarios were studied via logistic regression (Figure 5), those being the north-to-south expansion route or vice versa. The first scenario was the most probable and was estimated at 0.85 (CI 0.82–0.88), indicating colonisation range expansion from the Alpine region towards the south. 

## 4. Discussion

The 23 natural *Q. cerris* populations of this study show a high degree of diversity, as observed in most of the woody plants [44]: all eight analysed loci are polymorphic (no fixed alleles were detected), and genetic diversity, measured by heterozygosity, appears to be high. The average *H_e_* values obtained for the 23 populations studied are in the range of 0.65–0.85 and do not show a geographical trend. These values are in the same range as those observed in a set of European populations of *Q. petraea* and *Q. robur* by Muir and Schlötterer [2], for which *H_e_* values were registered between 0.3 and 0.96. The smaller range of variation in our populations is easily explained by the more restricted range analysed. The same effect is evident when comparing our results to those obtained by Streiff et al. [45] in the same species, using two of the SSR loci present in this work, MSQ4 and MSQ13, respectively. For the latter locus, *Q. cerris* displays lower *H_e_* levels (0.27) than *Q. robur* and *Q. petraea* (0.79 and 0.85). The high variability is not restricted to the nuclear genome; as shown by Simeone et al. [46], *Q. cerris* shows very high diversity both for plastid DNA and for the spacer of 5S RNA when compared with other oaks of the sect. *Cerris*.

The detected deviation from the Hardy–Weinberg equilibrium is almost exclusively associated with a significant deficit of heterozygotes. It is possible to explain the heterozygote deficiency observed in the present study by a selection against heterozygotes or by the presence of null alleles (possibly arising from mutations in the region of homology to the SSR primers), which could have led to an underestimation of *H*. The latter hypothesis is the most likely, since we used SSR primers developed in the congener species *Q. macrocarpa*, *Q. petraea* and *Q. robur* for our analysis. In particular, the primers derived from *Q. macrocarpa*, the most phylogenetically distant species, are those displaying, on average, the lowest *H_e_* values in our populations. The possibility that linkage disequilibrium has arisen as a consequence of physical linkage between the loci can be ruled out. This is because SSRs that mapped on different chromosomes in a composite *Fagaceae* map [25] were chosen. However, LD levels in our populations are not high, and thus, cannot be seen as footprint of a recent admixing event between populations, as also indicated by the analysis of the genetic structure discussed later. The presence of LD can also be diagnostic of a role as potential refugium for that particular population. In our work, the only population characterised by moderate LD is MG, whose geographic position makes it unsuited for the role. Another cause for LD is genetic drift; however, the number of SSRs used prevented us from testing for bottlenecks, the other characteristic for a refugium, since the most common tests rely on at least 20 loci to yield sound results [47]. However, the result of the Mantel test was not significant, thus corroborating the view that genetic drift has not been a major force shaping the genetic structure of the studied population.

### 4.1. Genetic Differentiation 

A significant level of genetic differentiation was found between the Italian populations of *Q. cerris*. The estimate, based on *F_ST_* = 0.054, is in the range already observed for other members of the Oak family; Muir and Schlötterer [2] observed *F_ST_* values between 0.001 and 0.232, average 0.05, between *Quercus petraea* and *Quercus robur* analysing 20 microsatellites in 12 populations. In the only other large-scale study conducted in Italy, five populations of *Q. petraea* analysed at five SSRs were characterised by *G_ST_* = 0.18 [48]. In oaks, the amount of genetic differentiation estimated by microsatellites appears higher than for other classes of genetic markers [1]; one of the markers exhibiting the highest differentiation in our study, QrZAG96 (*F_ST_* = 0.148), was an outlier also in *Quercus petraea* and *Quercus robur* [1,2]. As suggested by Lexer et al. [13], this indicates genomic heterogeneity in *Q. cerris*; a support to this possibly comes from the other “outlier” in our study—the heterologous marker MSQ13. This marker has in fact been developed in the more distant *Quercus macrocarpa* and, hence, has possibly been unwillingly selected for its higher ability to detect differentiation. Genetic variability at this locus is low and results in the presence of different homozygotes in the populations studied. While the latter is a marker known for displaying high differentiation values in oak populations [1,2], the former is the one displaying lower *He* values, based on heterologous primers devised in *Q. macrocarpa*. If we remove MSQ13 from the dataset, the overall estimate for genetic differentiation will be *F_ST_* = 0.059 (0.037 ≤ *F_ST_* ≤ 0.099).

The genetic divergence between populations was also estimated by *R_ST_*, the *F_ST_* analogue based on the stepwise mutation model. An overall value of *R_ST_* = 0.071 was found, indicating that the populations studied were more differentiated in the past due to the ability of *R_ST_* to detect differentiation events older than those revealed by *F_ST_*. Range expansion and increased gene flow have both contributed to the homogenisation of genetic differences among populations. Genetic differentiation was evaluated also between pairs of populations and proved significant in most cases, based on a permutation test. Further, in this case, *R_ST_* values are consistently higher than corresponding *F_ST_*. The lowest differentiation was found, concerning *F_ST_*, for the population pair AC–CV and BC–MA (*F_ST_* = 0.05), BF–LI (*F_ST_* = 0.01), and MT–SA (*F_ST_* = 0.00), and concerning *R_ST_*, for couples LA–ES (*R_ST_* = 0.02), MA–BC and CO–CF (*R_ST_* = 0.01), BE–BC, and CO–BA and MT–BR (*R_ST_* = 0.00).

What do these data tell us about the natural history of the species? It appears that the amount of genetic differentiation for *Q. cerris* is of the same order, albeit lower, than that experienced by other *Quercus* species. In the already cited paper by Curtu et al. [14], the overall genetic differentiation between four white oak species was *F_ST_* = 0.174 for isozymes and *F_ST_* = 0.092 for SSRs. According to Corti et al. [19], *Q. cerris* is native to South-central and Southeastern Europe; Fineschi and Vendramin [49] suggested for oaks, based on plastid data, a migration pattern in Italy going from south to north and, to a lesser extent, from east (Balkans) to west. A study on chloroplast SSRs [15] used fossil data to construct a demographic model for the species; this showed an expansion from the more diverse Anatolian region towards Central and Eastern Europe and then a further expansion into Northern Italy and the Balkans. The latter study conflicts with the former as the species seems to have expanded in opposite directions when involving the Italian populations. However, our demographic analyses showed a north-to-south gradient in Italy colonisation, confirming the more recent findings.

### 4.2. Genetic Structure

The detection of *K*, the most probable number of ancestral gene pools or ‘inferred populations’ from the used data, is of both conservationist and phylogeographic importance. This is usually obtained by means of a Bayesian approach, a methodology that has been increasingly popular in the last two decades [50,51]. In the present study, the number of ancestral homogeneous gene pools from which the studied populations derive was best estimated by *K* = 4. Each of these ancestral gene pools contributed in a different way to the twenty-three populations of *Q*. *cerris* sampled. 

When dealing with conservation and phylogeographic issues, it is often necessary to detect *K*, the number of panmictic units or ‘gene pools’ in the data, in order to be able to suggest possible mechanisms that have shaped the genetic variability observed. The use of a Bayesian approach to the detection of *K* has become increasingly popular in the last decade [50,51]. In the present study, it was possible to estimate *K* = 4 as the number of inferred populations from which the studied populations derive. The most precise interpretation of this value is that four homogeneous gene pools contributed to the twenty-three populations sampled. The distribution of the populations deriving their genetic makeup from the four pools, if not clear-cut, shows evidence of some geographic structuring, roughly northwest vs. southeast, with a zone of low admixture corresponding to a high degree with the valley of the Tiber river. It appears from these data that the colonisation of the Italian peninsula can be thought as having followed two routes; however, it is not possible yet to indicate their origin with an acceptable degree of certainty. The use of uniparentally inherited markers will allow us to further investigate this issue. Under this aspect, of particular interest could be the analysis of the scattered populations present in Southern France, because a reduced speed of re-colonisation for some European trees than previously estimated has been suggested [21]. If this is true, and some species do not have a complete coverage of potential habitat, a genetic analysis of the populations at the extreme boundary of the actual distribution will be helpful to understand more about post-glacial colonisation routes.

### 4.3. Demographic Expansion in Italy

The ABC approach allowed us to reliably estimate the posterior coefficient *r*_0_ (present/past genetic diversity), as indicated by the sharp posterior distributions of *r*_0_ and the values of the PABC index, which are, with the exception of the “North” one, comparable with those deemed acceptable in previous studies [42]. The gene pool showing the widest distribution is also the one that did not show footprints of demographic variation in the timeframe considered by the analysis. The mixture of stability and expansion signals shown by the ABC approach is compatible with the presence of a continuous forest, which has witnessed successive arrivals from several sources. A previous study on the demographic history of *Q. cerris* [15] dated divergence of Anatolian populations and Northern Balkan and Italy populations between 261 and 435 kya. The homogeneous gene pool of origin for populations MG, BA, BS and CO, and its recent expansion, seems to agree with the hypothesis of a southern Adriatic land bridge suggested by the same paper [15].

In general, it looks like *Q. cerris* found a favourable environment in the Italian peninsula, leading to an increase in forest cover for this species in the last few hundred millennia. It has to be borne in mind, however, that *Q. cerris* lives in sympatry with several other species of white oaks; therefore, a genetic analysis at the genomic level could shed more light on the ecology of this species complex.

### 4.4. Implication for Conservation and Further Work

The concept of “glacial refugia” has been recently challenged. Bennett and Provan [52] argued that this term has done its time and should be replaced by a more comprehensive description of the natural history of the species especially focused on their distribution and abundance through time. A way to do this is represented by a close examination of those populations (or sub-populations) that have most contributed to the range expansion of a given species after the Last Glacial Maximum. Besides these aspects, the genetic analysis at the population level still represents one of the most powerful tools for the discovery of populations, which, because of their position and history, could harbour genotypes not found elsewhere for a given species. This would represent a real bounty for the conservation of natural resources, since populations living in isolated regions and/or subject to particular environmental conditions could possibly be hiding unexplored useful genetic variants, the use of which can enhance the efficiency and value of conservation strategies. In this context, demographic analysis can also represent an important tool to describe fluctuations in the number of individuals for genetically homogeneous groups, leading to a reconstruction of the ecological history also. In this frame, the present work represents a step toward an integrated synthetic map of the genetic variability for an important oak species, thus laying the foundation for future studies aimed at conservation. Finally, the traditional concept of species has only rarely been challenged from the complex of species of the *Quercus* genu; so, it will be necessary to resort to a genetic analysis with higher resolution power to take into account the possible problems raised by sympatric species. 

## Figures and Tables

**Table 1:** Natural populations of *Quercus cerris* used in this study and their current management. Observed and expected heterozygosity measured at each locus for each population, and averages over loci and populations, are reported.

Population	Location	No. of Samples	Latitude N	Longitude E	*H_e_*	*H_o_*	Average No. Alleles/Locus	Forest Management
AC	Albarè di Costermano	30	45°33′59″	10°45′07″	0.697	0.662	10.6	High forest
BA	Bari	32	41°07′33″	16°52′06″	0.734	0.685	12.3	Wooded scrub
BC	Colli Berici	36	45°27′01″	11°32′19″	0.723	0.559	13.9	Coppice
BE	Bedonia	34	44°30′06″	9°38′02″	0.687	0.615	12.1	Coppice
BF	Bosco della Fontana	30	45°13′13″	10°45′25″	0.734	0.582	10.9	High forest
BR	Bagno di Romagna	30	43°50′00″	11°57′35″	0.716	0.621	13.6	Coppice
BS	Barisciano	40	42°19′31″	13°35′36″	0.712	0.587	12.5	Coppice
CF	Campofontana	30	45°38′21″	11°09′17″	0.720	0.675	13.5	Coppice
CM	Casale Monferrato	43	45°08′13″	08°27′03″	0.716	0.604	12.8	Young high forest
CO	Monte Conero	40	43°33′04″	13°36′16″	0.707	0.639	14.5	Coppice
CV	Cala Violina, Follonica	34	42°52′43″	10°49′43″	0.743	0.624	14.4	Coppice
DC	Dolegna del Collio	30	46°01′53″	13°28′45″	0.730	0.628	13.6	Young high forest
ES	Esenta	44	45°25′01″	10°28′51″	0.716	0.559	15.0	High forest
FI	Ficuzza	30	37°52′58″	13°22′37″	0.698	0.575	10.3	High forest
LA	Laveno, Monte Sangiano	30	45°54′37″	8°37′08″	0.717	0.539	11.6	High forest
LI	Ligonchio	30	44°18′57″	10°20′34″	0.758	0.582	12.8	Coppice
MA	Manziana	40	42°07′59″	12°07′38″	0.756	0.563	13.9	High forest
MD	Monghidoro	40	44°13′39″	11°19′43″	0.672	0.597	12.0	Coppice
MG	Mongiana	40	38°30′50″	16°19′13″	0.679	0.599	10.1	High forest
MT	Matera	37	40°40′07″	16°36′21″	0.805	0.503	12.3	Wooded scrub
SA	Sabaudia	36	41°17′59″	13°01′28″	0.794	0.508	11.9	High forest
VC	Vercelli	40	45°19′16″	8°25′34″	0.650	0.620	8.3	High forest
VT	Val Trompia, Lodrino	33	45°43′11″	10°16′36″	0.803	0.604	15.1	Coppice
			Average/population		0.725	0.597		

**Table 2:** Features of the microsatellite markers used in this study.

SSR Name	Repeat	Primer Sequence (5′–3′)	*T_a_* (°C)	Fluorophore Used	Number of Detected Alleles	Sizes of Alleles (bp)
*Qp*ZAG9	(AG)12	**F**: GCAATTACAGGCTAGGCTGG **R**: GTCTGGACCTAGCCCTCATG	55 °C	HEX	40	175–281
*Qp*ZAG110	(AG)15	**F**: GGAGGCTTCCTTCAACCTACT **R**: GATCTCTTGTGTGCTGTATTT	53 °C	FAM	33	200–272
*Qr*ZAG4	(GA)46	**F**: CGTCTATAAGTTCTTGGGTGA **R**: GTAACTATGATGTGATTCTTACTTCA	50 °C	FAM	37	99–173
*Qr*ZAG7	(TC)17	**F**: CAACTTGGTGTTCGGATCAA **R**: GTGCATTTCTTTTATAGCATTCAC	51 °C	HEX	35	101–169
*Qr*ZAG20	(TC)18	**F**: CCATTAAAAGAAGCAGTATTTTGT **R**: GCAACACTCAGCCTATATCTAGAA	50 °C	TAMRA	38	126–210
*Qr*ZAG96	(TC)20	**F**: CCCAGTCACATCCACTACTGTCC **R**: GGTTGGGAAAAGGAGATCAGA	55 °C	TAMRA	20	136–176
MSQ4	(GA)17	**F**: TCTCCTCTCCCCATAAACAGG **R**: GTTCCTCTATCCAATCAGTAGTGAG	49 °C	FAM	38	115–253
MSQ13	(GA)14	**F**: TGGCTGCACCTATGGCTCTTAG **R**: ACACTCAGACCCACCATTTTTCC	53 °C	HEX	24	189–249

**Table 3:** Estimated demographic parameters for each ancestral pool. Brackets indicate 95% confidence intervals. *θ*_0_, effective population size before event. *θ*_1_, effective population size after event. r_0_, coefficient of *θ*_0_*/θ*_1_. T, time in generations.

	θ_0_	θ_1_	r_0_	T
Pop1	38.20 [1.99–91.50]	0.09 [0.01–0.76]	424.44	4800 [57–9950]
Pop2	36.20 [1.57–90.20]	0.08 [0.01–0.76]	452.50	3320 [32–9900]
Pop3	23.20 [0.82–84.20]	0.59 [0.01–7.87]	39.32	972 [14–9490]
SIC/Pop4	8.99 [0.30–67.80]	5.87 [1.36–26.10]	1.53	628 [17–9090]

## Data Availability

Genotyping data are available from the Authors.

## Supplementary Materials

The following supporting information can be downloaded at: https://www.mdpi.com/article/10.3390/life13010018/s1, Figure S1: *Quercus cerris* distribution range; Figure S2: Barplot from the Bayesian analysis of population structure; Table S1: *F_ST_* & *R_ST_*. *F_ST_* (above diagonal) and *R_ST_* (below diagonal) values for each population pair; Table S2: (a) DIC values and relative standard deviations for each K tested and test of significance. See text for details. (b) Test of significance for the different *K* values; Table S3: PABC index (see Section 2) calculated for the demographic scenarios proposed for the four gene pools.
