# Supplementary material for: Genetic Variability and Admixture Zones in the Italian Populations of Turkey Oak (Quercus cerris L.)
Source: Life (Basel). 2022 Dec 21;13(1):18. doi: 10.3390/life13010018 (PMC9863001; doi:10.3390/life13010018)
Supplement: Supplementary file 1 [file life-13-00018-s001.zip › life-2047762-supplementary.pdf]

SUPPLEMENTARY MATERIALS

Table S1. F_{ST} & R_{ST}. F_{ST} (above diagonal) and R_{ST} (below diagonal) values for each population pair. Values in bold do not significantly differ from zero (P<0.05), according to the results of a permutation test.

	AC	BC	BA	BE	BF	BR	BS	CF	CM	CO	CV	DC	ES	FI	LA	LI	MA	MD	MG	MT	SA	VC	VT
AC	-	0.051	0.087	0.026	0.048	0.050	0.088	0.072	0.012	0.083	**0.005**	0.009	0.026	0.075	0.009	0.049	0.044	0.079	0.127	0.041	0.022	0.105	0.043
BC	0.048	-	0.099	0.014	0.095	0.071	0.100	0.088	0.045	0.097	0.047	0.044	0.034	0.089	0.052	0.087	**0.005**	0.101	0.114	0.060	0.054	0.130	0.068
BA	0.154	0.128	-	0.090	0.043	0.031	0.019	0.043	0.096	0.009	0.086	0.076	0.043	0.048	0.099	0.045	0.069	0.053	0.065	0.047	0.061	0.100	0.060
BE	0.024	0.000	0.125	-	0.076	0.064	0.093	0.084	0.013	0.091	0.026	0.024	0.011	0.097	0.020	0.077	0.017	0.078	0.121	0.049	0.041	0.111	0.057
BF	0.090	0.056	0.116	0.043	-	0.016	0.059	0.028	0.054	0.042	0.052	0.046	0.050	0.037	0.058	**0.001**	0.069	0.063	0.099	0.016	0.016	0.093	0.030
BR	0.056	0.004	0.106	0.014	0.036	-	0.034	0.013	0.057	0.022	0.048	0.030	0.030	0.010	0.066	0.012	0.046	0.057	0.075	0.025	0.026	0.100	0.040
BS	0.165	0.153	0.004	0.158	0.171	0.131	-	0.042	0.102	0.014	0.090	0.070	0.050	0.050	0.103	0.049	0.071	0.052	0.063	0.058	0.061	0.103	0.072
CF	0.140	0.118	0.007	0.118	0.081	0.061	0.033	-	0.081	0.037	0.067	0.052	0.052	0.031	0.077	0.017	0.060	0.070	0.082	0.035	0.041	0.124	0.043
CM	0.039	0.045	0.145	0.015	0.031	0.067	0.186	0.151	-	0.092	0.013	0.014	0.024	0.086	0.015	0.057	0.042	0.088	0.131	0.034	0.022	0.116	0.044
CO	0.152	0.132	0.000	0.132	0.126	0.100	0.020	0.001	0.163	-	0.080	0.057	0.045	0.042	0.098	0.039	0.065	0.057	0.074	0.056	0.054	0.112	0.064
CV	0.020	0.084	0.114	0.051	0.084	0.082	0.130	0.125	0.028	0.122	-	0.008	0.026	0.069	0.013	0.051	0.041	0.072	0.119	0.027	0.020	0.106	0.045
DC	0.054	0.003	0.127	0.025	0.082	0.001	0.014	0.120	0.084	0.127	0.075	-	0.015	0.063	0.015	0.041	0.034	0.066	0.116	0.029	0.009	0.107	0.043
ES	0.016	0.036	0.121	0.009	0.026	0.027	0.153	0.091	0.007	0.120	0.012	0.053	-	0.056	0.027	0.047	0.024	0.042	0.091	0.028	**0.027**	0.086	0.045
FI	0.076	0.081	0.072	0.076	0.074	0.039	0.072	0.034	0.109	0.088	0.079	0.055	0.069	-	0.099	0.030	0.073	0.084	0.069	0.048	0.048	0.127	0.055
LA	0.028	0.050	0.059	0.009	0.028	0.044	0.090	0.048	0.004	0.081	0.009	0.071	0.002	0.027	-	0.062	0.052	0.079	0.135	0.038	0.027	0.114	0.045
LI	0.087	0.089	0.136	0.073	0.016	0.067	0.165	0.101	0.046	0.162	0.070	0.102	0.042	0.042	0.021	-	0.061	0.055	0.088	**0.009**	0.017	0.096	0.034
MA	0.025	0.001	0.123	0.007	0.093	0.016	0.130	0.110	0.061	0.122	0.066	0.012	0.035	0.072	0.043	0.109	-	0.083	0.103	0.039	0.034	0.113	0.055
MD	0.119	0.090	0.096	0.099	0.040	0.028	0.121	0.028	0.123	0.088	0.111	0.068	0.068	0.029	0.077	0.052	0.105	-	0.107	0.045	0.065	0.018	0.068
MG	0.302	0.304	0.133	0.328	0.277	0.249	0.109	0.150	0.344	0.131	0.254	0.253	0.286	0.163	0.262	0.264	0.296	0.151	-	0.095	0.094	0.148	0.097
MT	0.064	0.032	0.098	0.031	0.022	0.000	0.123	0.041	0.062	0.098	0.064	0.026	0.018	0.022	0.026	0.032	0.041	0.008	0.228	-	**0.000**	0.079	0.012
SA	0.054	0.047	0.174	0.048	0.064	0.026	0.183	0.133	0.060	0.183	0.048	0.042	0.020	0.059	0.049	0.038	0.047	0.062	0.303	0.008	-	0.102	0.018
VC	0.126	0.111	0.156	0.122	0.052	0.057	0.180	0.084	0.144	0.157	0.159	0.103	0.098	0.039	0.102	0.051	0.133	0.014	0.218	0.043	0.098	-	0.095
VT	0.003	0.093	0.180	0.077	0.126	0.082	0.178	0.151	0.086	0.166	0.022	0.079	0.036	0.096	0.076	0.113	0.058	0.118	0.270	0.078	0.053	0.137	-

Table S2. (a) DIC values and relative standard deviations for each K tested and test of significance. See text for details. (b) Test of significance for the different *K* values.

a.

K	DIC	s.d.
2	65407.3	209.2
3	64671.3	64.8
4	64243.0	175.8
5	64033.9	203.3
6	63872.9	502.0
7	63744.3	234.5
8	63656.0	243.9
9	63616.7	294.8
10	63595.4	254.4

b.

	t	p	Standard Bonferroni
K2 vs K3	14.8	6.35E-08	5.08E-07
K3 vs K4	11.95	3.99E-07	3.19E-06
**K4 vs K5**	**5.03**	**0.000356**	**2.85E-03**
K5 vs K6	1.56	0.07619	6.10E-01
K6 vs K7	1.45	0.09016	7.21E-01
K7 vs K8	7.02	3.09E-05	2.48E-04
K8 vs K9	1.15	0.1401	1.00E+00
K9 vs K10	0.51	0.3115	1.00E+00

Table S3. PABC index (see Section 2) calculated for the demographic scenarios proposed for the four gene pools.

		PABC
	North	0.125
	Central	0.372
	South	0.394
	Sicily	0.360

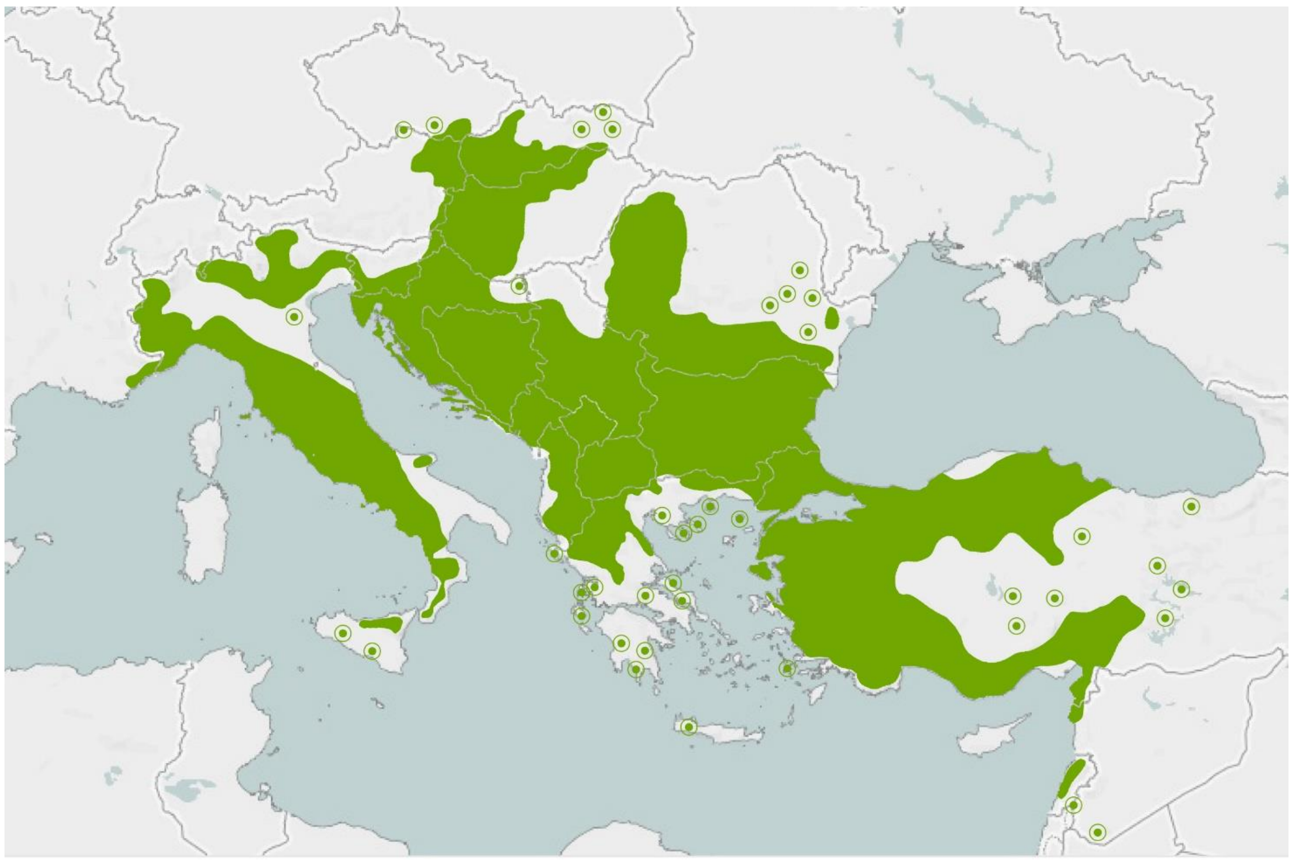

Figure S1. *Quercus cerris* distribution range. The native distribution range of the Turkey oak is shown in green while green circles represent isolated populations as described in Caudullo et al. [1] from which this map was modified from.

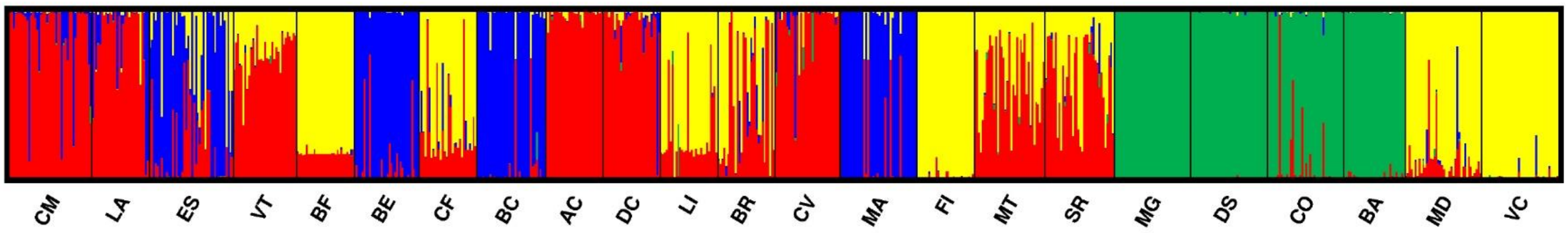

Figure S2. Barplot from the Bayesian analysis of population structure. Each bar represents a tree; colours indicate the proportion of genome from each of the four ancestral gene pools. Populations are indicated according to the code of Table 1 in the text.

References

1. Caudullo G.; Welk E.; San-Miguel-Ayanz J. Chorological maps for the main European woody species. *Data Brief* **2017**, 12:662-666.
